# The Association of Systemic Inflammatory Response with Hepatitis B Vaccine Unresponsiveness

**DOI:** 10.3390/v17030338

**Published:** 2025-02-28

**Authors:** Oguz Karabay, Kaan Furkan Hamarat, Gamze Guney Eskiler, Ayhan Aydin

**Affiliations:** 1 Department of Infectious Diseases and Clinical Microbiology, Faculty of Medicine, Sakarya University, 54290 Sakarya, Türkiye; 2 Faculty of Medicine, Sakarya University, 54290 Sakarya, Türkiye; hamaratkaanfurkan@gmail.com; 3 Faculty of Medicine, Department of Medical Biology, Sakarya University, 54290 Sakarya, Türkiye; gamzeguney@sakarya.edu.tr; 4 Department of Internal Sciences, Sakarya University Training and Research Hospital, 54290 Sakarya, Türkiye; doctorayhanaydin@gmail.com

**Keywords:** Hepatitis B virus, inflammatory parameters, immune response, systemic inflammation, vaccine response

## Abstract

(1) Background: Hepatitis B virus (HBV) infection remains a major health challenge. Although vaccination protects people from HBV infection, 5–10% of people at risk of HBV infection and associated liver diseases do not respond to vaccination. The association of hematological indices with vaccine response is a crucial contributing factor in HBV-associated liver damage and the outcome of patients. In this context, we clinically assessed the interaction between inflammatory parameters and Hepatitis B vaccine response for the first time. (2) Methods: In total, 90 volunteers (44 non-responders and 46 responders) were included in this retrospective study. The demographic data and the hemogram parameters of the volunteers were recorded and statistically analyzed. Additionally, systemic inflammation index (SII), platelet-to-lymphocyte ratio (PLR), and neutrophil-to-lymphocyte ratio (NLR) were calculated. (3) Results: Our results indicate that higher median levels of white blood cells (8.61), lymphocytes (2.37), neutrophils (5.71), and platelets (280) were determined in the non-responders compared to the responders. SII and NLR indices were significantly higher in the non-responders than in the responders (*p* < 0.05). (4) Conclusions: The non-responders exerted higher systemic inflammation indicators than the responders, and the NLR value partially distinguished Hepatitis B vaccine response. Nevertheless, further studies with larger cohorts are essential to confirm the clinical utility of systemic inflammatory response as a reliable criterion for predicting Hepatitis B vaccine responsiveness.

## 1. Introduction

Hepatitis B virus (HBV) infection is still a worldwide health problem, with an estimated 1.5 million new infections every year, leading to chronic hepatitis, cirrhosis, and hepatocellular carcinoma. In 2019, Hepatitis B infection caused an estimated 820,000 deaths [1].

Vaccination decreases the risk of Hepatitis B infection. However, immune response to the HBV vaccine fails in 5–10% of individuals. Some diagnostic mechanisms such as antigen presentation failure, polymorphisms of human leukocyte antigen (HLA) class II alleles, changes in the gene expressions associated with immune regulation or lack of specific CD4 T helper (Th) cells as well as gender, age, obesity, and smoking play a crucial role in Hepatitis B vaccine non-responsiveness [2,3,4]. Nonetheless, the underlying immunological mechanisms remain unclear. Therefore, further clinical studies should be performed to elucidate the association of underlying molecular mechanisms and clinical features with Hepatitis B responsiveness and to improve immunogenicity for overcoming non-responsiveness.

Systemic inflammation plays a role in different diseases such as chronic liver disease, fibrosis, and hepatocellular carcinoma [5,6,7,8,9,10]. The systemic inflammatory response is assessed using the systemic inflammation index (SII), neutrophil-to-lymphocyte ratio (NLR), and platelet-to-lymphocyte ratio (PLR) to non-invasively predict HBV infection-related liver disease and different types of tumors. Hu et al. [11] developed the SII to predict the prognosis of hepatocellular carcinoma patients after curative resection. SII’s cut-off value is suitable for predicting prognosis in all cancer patients, as well as cerebrovascular and cardiovascular diseases and rheumatoid arthritis [11,12]. The NLR can act as a marker for subclinical inflammation and is associated with the innate immune response regulated by neutrophils (NEU) and lymphocytes (LYMP). The PLR, based on platelet aggregation and systemic inflammation, evaluates platelet activation triggered by systemic inflammatory or inflammatory–coagulation reactions. In this context, SII could potentially indicate the relationship between the inflammatory response and immune status [13,14]. In HBV associated diseases, there is a significant relationship between NLR and PLR levels and fibrosis, and PLR and NLR levels could play role in managing chronic HBV infection related to liver disease [6,10]. However, the clinical significance of systemic inflammatory indicators, especially the NLR and PLR in Hepatitis B vaccine response, remains unclear.

In this context, we, for the first time, investigated the relationships between inflammatory parameters and their associated indicators and Hepatitis B vaccine response.

## 2. Materials and Methods

### 2.1. Subjects

This retrospective study was conducted at Sakarya University, Faculty of Medicine, Department of Infectious Diseases and Clinical Microbiology, between June 2019–December 2020. A total of 90 healthy volunteers, including 44 non-responders and 46 responders, were selected, and each group was matched according to age and sex. The population of our study consisted of healthy individuals who applied to the vaccination polyclinic within the Infectious Diseases clinic at Sakarya University. These individuals were selected from people who did not have any acute or chronic infectious diseases and were seronegative for Hepatitis B virus (HBV) infection. Participants generally consisted of individuals in the middle-age group. They worked in the university (academic and administrative) and other segments of the hospital. A history of allergies or severe reaction to vaccination, previous immune-suppressive or immunostimulation therapy, acute illness within the past 7 days, HIV-positive individuals, and pregnant women were excluded in this study. The volunteers negative for Hepatitis B infection agreed to participate. Additionally, participants were retested for Hepatitis B and C infections and confirmed to be negative for these infections. HIV-positive individuals and those suffering from acute illness were not included in the study. All participants were vaccinated with three doses (0, 1, and 6 months) of the Hepatitis B vaccine (rDNA) (Serum Institute of India, India), and anti-HBs titers were analyzed 1 month after the last dose. Each dose of 0.5 mL contains 10 mcg of Hepatitis B surface antigen adsorbed on 0.25–0.40 mg aluminum hydroxide. The anti-HB levels (2–1000 mIU/mL and >1000 mIU/mL) were analyzed using an Architect anti-HBs assay (Abbott Laboratories, Dublin, Ireland). The measuring interval of the anti-HB assay is 2.00 mIU/mL to 1000 mIU/mL. Limit of detection (LoD) and limit of quantitation (LoQ) values were 0.77 mIU/mL and 2 mIU/mL, respectively. Non-responders with anti-HB titers were categorized as <10 mIU/mL. Responders had received a three-dose standard regimen and appropriate anti-HB titers (>10 mIU/mL). The Ethical Committee approved this study at Sakarya University, Faculty of Medicine (Number: E-71522473-050.01.04-15096-134), and it was conducted according to the principles expressed in the Declaration of Helsinki.

### 2.2. Analysis of Inflammation Indicators

To determine the status of inflammation-associated indicators in the responders and the non-responders, leukocyte count (WBC), platelet count (PLT), NEU, and LYMP count were collected. C-reactive protein (CRP) values were collected by Marc Buncher, Siemens. CRP values are <0.3 mg/dL: Normal; 0.3–1.0 mg/dL: Normal or minor elevation; 1.0–10.0 mg/dL: Moderate elevation (Systemic inflammation), >10.0 mg/dL: Marked elevation and more than 50.0 mg/dL: Severe elevation [15]. Additionally, we calculated the SII (SII = neutrophil count X platelet count/lymphocyte count), PLR (PLT/LYMP), and NLR (NEU/LYMP) values.

### 2.3. Statistical Analysis

Statistical analysis was performed using SPSS 22.0 (SPSS Inc., Chicago, IL, USA), and significance was accepted at a *p* < 0.05. Nonparametric statistical tests were performed in all cases. Data were analyzed categorically for the antibody titer. The Mann–Whitney U test was used for comparison. A Spearman correlation coefficient was used to assess the relation systemic inflammation parameters with Hepatitis B vaccine response. A multivariate diagnosis model was constructed using binary logistic regression. ROC curves were used to determine the predictive importance of PLR and NLR for vaccine response, and cut-off values were analyzed by calculating the area under the ROC curves (AUC).

## 3. Results

### 3.1. Demographic Features of Subjects

This study included 44 non-responders and 46 responders. Of the total participants, 22 females and 22 males did not develop a sufficient anti-HB response and were called non-responders. Additionally, 21 females and 18 males had an anti-HB titer between 10 and 1000 mIU/mL, and 3 females and 4 males had an anti-HB titer >1000 mIU/mL. In total, 24 females and 22 males were classified as responders. The age range of the non-responders was 38–48 and 36–54 years for females and males, respectively, whereas the age range of the responders was 37–44 and 41–51, respectively.

### 3.2. Evaluation of Systemic Inflammation

To compare systemic inflammation status in the responders and non-responders, we assessed CRP, WBC, PLT, NEU, and LYMP values. As shown in Table 1, higher levels of WBC (8.61, *p =* 0.00), LYMP (2.37, *p =* 0.09), NEU (5.71, *p =* 0.00), and PLT (280, *p =* 0.03) were observed in the non-responders compared to the responders (WBC: 6.45, LYMP: 2.07, NEU: 3.73, PLT: 241). Additionally, the CRP level was significantly higher in the non-responders (6.81) than in the responders (3.20) (*p =* 0.03), as seen in Table 1. Furthermore, the SII (*p =* 0.04) and NLR (*p =* 0.01) values were significantly different between the non-responders and the responders.

The correlation analysis revealed that a significant correlation between WBC, LYMP, NEU, PLR, and NLR values and SII in the non-responders (Table 2). In contrast, there was a considerable correlation between NEU, PLT, PLR, and NLR values and SII in the responders. Furthermore, a significant positive correlation was analyzed between LYMP, NEU, PLT, CRP, and WBC levels in the non-responders (*p* < 0.05). There was a negative significant correlation between anti-HBs and PLT in the non-responders (*p* < 0.05).

In the binary logistic regression model (Table 3), the NLR value was a significant predictor of Hepatitis B vaccine response (*p* < 0.05). A correct classification rate of 63.4% was obtained for PLR and NLR in Hepatitis B vaccine response.

**Figure 1:**
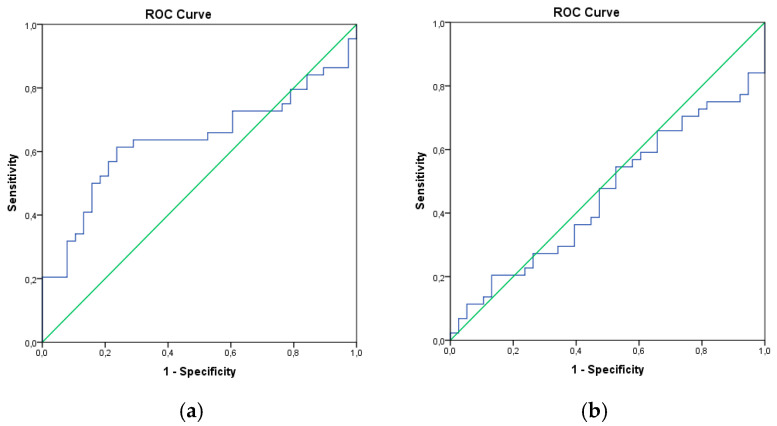
The histograms of the ROC curve analysis. We analyzed the discrimination of Hepatitis B vaccine response according to (**a**) NLR and (**b**) PLR parameters. The ROC curve results were used to evaluate an optimal cut-off value for NLR and PLR determining Hepatitis B vaccine response.

Furthermore, these findings were supported by the ROC curve analysis (Figure 1). The cut-off values of NLR and PLR were 2.05 (*p* < 0.05) and 119.06 (*p* > 0.05), respectively (Table 4). Consequently, NLR could predict Hepatitis B vaccine response.

## 4. Discussion

To our knowledge, this is the first study to clinically assess the association of systemic inflammation status through clinical parameters (CRP, WBC, PLT, NEU, and LYMP levels) with Hepatitis B vaccine response. Our findings indicated that the non-responders exerted higher systemic inflammation indicators than the responders due to higher CRP, WBC, PLT, NEUT and LYMP levels and SII and NLR indices.

Indeed, some limited studies have investigated the association of changes in cytokines, chemokines, and genes with Hepatitis B infection [2,4,16]. Körber et al. noted that an increased CD24_high_CD38_high_ regulatory B cells (Breg) mediating B and T cell responses are detected in the non-responders [4]. Therefore, anti-HB seroconversion is related to decreased Breg numbers after booster immunization [4]. Furthermore, IFN-gamma (IFN-γ), Interleukin (IL)-2, TNF-α, IL-12, and IL-10 and IL-4 levels are analyzed in seven non-responders, ten hypo-responders, and ten high-responders after in vitro stimulation of the peripheral blood mononuclear cells (PBMC) with recombinant HBV surface antigens (HBsAgs). They stated that the levels of these cytokines are decreased in the non-responders due to possibly insufficient or lack of T helper cells (Th)1 and Th2 responses [2]. In the study by Qui et al. [16], the expression patterns of genes, Th1/Th2/Th9/Th17/Th22/Treg cytokine, and chemokine profiles were analyzed using microarray analysis and a Luminex assay in the PBMCs of Hepatitis B vaccine non-responders and responders at five different time points (pre-vaccination, 3rd, 7th, and 28th post day following the first dose vaccination). They state that nine coding gene levels were increased in the non-responders at all five-time points with lower IL-27 and CXCL12 concentrations on the third day after the first dose and seventh day after the second dose [16]. However, no study has evaluated the relationship between hematologic parameters and Hepatitis B vaccine response. Our study detected higher WBC, PLT, NEU, and LYMP levels in the non-responders. T cell mediates the systemic response to infection by producing different cytokines. Th1 lymphocytes generate IFN-γ and IL-2, whereas Th2 cells produce IL-4, IL-5, IL-10, and IL-13 [17]. Therefore, elevated LYMP, WBC, and NEU levels in the non-responders could be associated with insufficient or a lack of Th1 and Th2 responses and a defect in the primary HBsAg-specific T-cell repertoire or antigen presentation. In this context, further investigations should be required for the identification of especially Th1- and Th2-mediated cytokine levels in responders and non-responders.

Systemic inflammation indicators, including NLR, PLR, and SII, play a crucial role in inflammatory response [5,6,7,8,9,10]. Konur et al. (2021) states that there is a special relationship between fibrosis and NLR and PLR in 173 patients with chronic HBV patients [6]. These findings are supported by Zhao’s (2017) study. In this study, the PLR and NLR are associated with the serum HBsAg levels and HBV infection-related liver disease in 172 chronic HBV-infected patients compared to the control [10]. However, there is no study evaluating the role of the PLR and NLR in Hepatitis B vaccine response. In our study, higher NLR and the SII values were found in the non-responders than the responders. However, there is no significant difference between the PLR and Hepatitis B vaccine response. NEU and LYMP mediate inflammatory processes, and changes in the number of NEU and LYMP are associated with inflammation [6,10,18,19]. Additionally, NLR could be a potential diagnostic or prognostic index in HBV-associated diseases such as fibrosis, hepatocellular carcinoma, and non-alcoholic fatty liver disease [6,10,18,19]. NLR is widely used for the immunological status of patients because NEU and LYMP in the peripheral blood are routinely controlled during clinical practice. Indeed, a lower NLR value is related to better clinical outcomes in Hepatitis B-related liver cirrhosis and hepatocellular carcinoma [6,10,19,20,21]. Kekilli et al. (2015) state that reduced levels of the N/L ratio can predict liver fibrosis in chronic hepatitis-infected patients [21]. Ren et al. (2020) states that higher SII, PLR, and NLR values are significantly related to poor prognosis in HBV-related hepatocellular carcinoma patients after liver transplantation [7]. On the other hand, there is no significant difference between the NLR value and fibrosis in chronic Hepatitis B patients [6,22]. Ding et al. (2021) stated that PLR potentially predicts advanced fibrosis and cirrhosis. However, the NLR level could not predict the inflammation and the amount of the accumulated fibrous tissue in the liver [23]. In our study, the non-responders had significantly higher NLR and SII values. Therefore, these values could predict the risk of Hepatitis B infection-associated disease in the non-responders. Additionally, lower LYMP rates could increase the NLR value, and leading to inadequate antibody response. In this context, higher NLR values may be a negative predictor in Hepatitis B vaccine response. However, a correct classification rate of both the PLR and NLR was lower for the prediction of Hepatitis B vaccine response. Thus, further studies should verify the predictive roles of the NLR, PLR, and SII values in a large number of non-responder volunteers. Additionally, we performed in single-center retrospective study. Therefore, our findings should be supported by multicenter prospective investigations with large-scale populations including healthy peoples and those with HBV-associated diseases.

Furthermore, the CRP level was higher in the non-responders compared to the responders in our study. In the study by Sing et al. (2020), the pooled mean level of CRP was high in patients with Hepatitis B compared to Hepatitis C in the meta-analysis. Thus, the pooled mean CRP level could determine the level of liver damage in patients with viral Hepatitis [24]. Gidado et al. (2022) stated that the presence of HBV infection is not predicted only using CRP level. However, high CRP levels in the blood can be a marker of inflammation [25]. In this context, high CRP levels could be associated with inflammation. However, only CRP level did not predict Hepatitis B vaccine response due to no significant results using binary logistic regression and ROC curve analyses. In this context, CRP could be elevated during acute inflammation and may be less sensitive in cases of chronic or low-grade inflammation. Additionally, individual differences affect CRP level. Therefore, further investigations should be required for the identification of the relationship between CRP level and Hepatitis B vaccine response.

## 5. Conclusions

In the present study, we assessed the association of systemic inflammation status, including NLR, PLR, and SII, with clinical parameters (CRP, WBC, PLT, NEU, and LYMP levels) to predict Hepatitis B vaccine response. Our results suggest that the non-responders had higher systemic inflammation indicators, including WBC, NEU, and PLT levels. Furthermore, the NLR value could be used as a biomarker for the risk of Hepatitis B infection-associated disease and for the prediction of Hepatitis B vaccine response in the non-responders and Hepatitis B vaccine response compared to the responders. However, there is a need for prospective studies with more non-responders and HBV-related patients to understand the association of these parameters with Hepatitis B vaccine response and the risk of advanced disease. In the future, systemic inflammatory indices, including SII, NLR, and PLR as simple and rapid test markers, will be used for vaccine response and the prediction of the risk HBV-associated disease.

## Figures and Tables

**Table 1:** Comparison of demographic and laboratory values in the non-responders and the responders (*p* < 0.05 *, *p* < 0.01 **).

Parameters	Non-Responders (*n* = 44) Median (Min-Max)	Responders (*n* = 46) Median (Min–Max)	*p*
Age			
Female	(38–48)	(37–44)	0.69
Male	(36–54)	(41–51)	0.95
WBC (4.0–10.0 K/μL)	8.61 (8.00–10.22)	6.45 (5.94–7.23)	0.00 **
LYMP (0.8–4 K/μL)	2.37 (2.14–2.81)	2.07 (1.80–2.42)	0.09
NEU (2.0–7.0 K/μL)	5.71 (4.55–6.27)	3.73 (3.46–3.99)	0.00 **
PLT (150–400 K/μL)	280 (261–294)	241 (218–266)	0.03 *
CRP (0.15–224 mg/L)	6.81 (3.30–11.18)	3.20 (3.32–4.00)	0.03 *
SII	552.51 (507.50–757.65)	455.83 (397.12–524.23)	0.04 *
PLR	118.59 (105.69–128.64)	119.57 (102.62–140.61)	0.51
NLR	3.39 (2.98–4.08)	2.032 (1.95–2.46)	0.01 **

**Table 2:** Spearman correlation of Anti-HBs, WBC, LYMP, NEU, PLT, and CRP levels and SII, PLR, and NLR values in the non-responders (NR) and responders (R) (*p* < 0.05 *, *p* < 0.01 **).

		LYMP	NEU	PLT	CRP	SII	PLR	NLR	Anti-HBs
**WBC**	**NR**	0.428 **	0.796 **	0.298 *	0.343 *	0.385 **	−0.302 *	0.077	−0.157
	**R**	0.764 **	0.871 **	0.263	0.173	0.292	−0.496 **	0.221	0.029
**LYMP**	**NR**		0.182	0.442 **	0.025	−0.432 **	−0.693 **	−0.638 **	−0.106
	**R**		0.429 **	0.310	0.066	−0.218	−0.682 **	−0.389 *	0.116
**NEU**	**NR**			0.132	0.362 *	0.523 **	−0.162	0.527 **	−0.113
	**R**			0.245	0.259	0.611 **	−0.201	0.586 **	−0.025
**PLT**	**NR**				0.094	0.269	0.177	−0.260	−0.345 *
	**R**				0.124	0.499 **	0.422 **	0.045	0.004
**CRP**	**NR**					0.143	−0.039	0.236	−0.279
	**R**					0.259	0.173	0.183	0.374
**SII**	**NR**						0.669 **	0.661 **	−0.171
	**R**						0.585 **	0.856 **	−0.039
**PLR**	**NR**							0.449 **	−0.106
	**R**							0.434 **	−0.079
**NLR**	**NR**								0.007
	**R**								−0.092

**Table 3:** Results of binary logistic regression. We assessed the association of PLR and NLR indices with Hepatitis B vaccine response (*p* < 0.05 *).

Parameters	Odds Ratio	95% CI	*p*
PLR	1.1	1.00–1.02	0.10
NLR	0.5	0.24–0.88	0.02 *

**Table 4:** The ROC curve results of NLR and PLR levels in the discrimination of non-responders versus responders using an Area Under Curve (AUC) analysis. This analysis provides the separation of NLR and PLR values to discriminate the accuracy of multivariate risk scores and categorize individuals as responders/non-responders (* *p* < 0.05).

	AUC	*p*	95% CI	Cut-Off	Sensitivity	1-Spesifity
NLR	0.635	0.036 *	0.512–0.759	2.05	0.636	0.368
PLR	0.458	0.509	0.332–0.583	119.06	0.477	0.526

## Data Availability

All data from this manuscript is available upon request by contacting Oguz Karabay.
